# Transferring Data from Smartwatch to Smartphone through Mechanical Wave Propagation

**DOI:** 10.3390/s150921394

**Published:** 2015-08-28

**Authors:** Seung-Chan Kim, Soo-Chul Lim

**Affiliations:** Device & System Research Center, Samsung Advanced Institute of Technology, 130 Samsung-ro, Yeongtong, Suwon, Gyeonggi-do 443-803, Korea; E-Mail: sc12.kim@samsung.com

**Keywords:** ultrasonic, acoustic transmission, non-radio communication, intra-body propagation, inter-device communication

## Abstract

Inspired by the mechanisms of bone conduction transmission, we present a novel sensor and actuation system that enables a smartwatch to securely communicate with a peripheral touch device, such as a smartphone. Our system regards hand structures as a mechanical waveguide that transmits particular signals through mechanical waves. As a signal, we used high-frequency vibrations (18.0–20.0 kHz) so that users cannot sense the signals either tactually or audibly. To this end, we adopted a commercial surface transducer, which is originally developed as a bone-conduction actuator, for mechanical signal generation. At the receiver side, a piezoelement was adopted for picking up the transferred mechanical signals. Experimental results have shown that the proposed system can successfully transfer data using mechanical waves. We also validate dual-frequency actuations under which high-frequency signals (18.0–20.0 kHz) are generated along with low-frequency (up to 250 Hz) haptic vibrations. The proposed method has advantages in terms of security in that it does not reveal the signals outside the body, meaning that it is not possible for attackers to eavesdrop on the signals. To further illustrate the possible application spaces, we conclude with explorations of the proposed approach.

## 1. Introduction

As wearable devices are becoming prevalent, data transfer between devices is becoming frequent and important. Previous work on personal area networks (PAN) pointed out the importance of a proper communication method between devices by describing that multiple devices without communication capability are prone to raise the issues of functional I/O redundancy [1]. For example, a person might carry multiple displays such as a smartwatch, mobile phone, and laptop, among others, if each device operates only for itself. In the case of recent smart devices, e.g., smartwatch and smartphone, each of these devices might need to be equipped with biometric sensors for the purpose of user authentication on each device. A variety of wireless technologies, such as near-field communication (NFC), Bluetooth, and infrared data association (IrDA) communications, have been proposed to resolve such redundancy issues. In the research area, many kinds of PAN, a small body-surrounding area where a set of specific devices can communicate with each other, have been proposed. Many of these systems establish connections by transmitting radio signals through the air. Although the wireless approach gives users great convenience, there can be issues involving message interception and vulnerabilities, including content spoofing [2]. This issue of data confidentiality may be mitigated by establishing communication channels without revealing signals to the air - *wired* communication. In the context of PAN, wired communication is often implemented by regarding the human body as an electrical wire by utilizing the fact that the human body is electrically conductive (see Section 2.1.1). However, some users are concerned about using their bodies as a part of an electrical circuit all the time, even though passing a small amount of time-varying electric current through the body is known to be safe [3,4,5]. These psychological concerns are attributed to the fact that wearable devices continuously maintain physical contact with the user’s body parts.

In the present study, we propose a novel inter-device communication system that transmits data through internal body structures using mechanical wave propagation. With the proposed approach, a smartwatch wrapped on the wrist can send signals to the fingertip such that the signals can be detected by a touch smartphone. The main contributions of the present study are:

(1) Use of mechanical waves through the human body parts for inter-device communication.
(2) Measurement and analysis of body-transmitted high-frequency signals.

## 2. Related Work

This section reviews previous work on on-body communication and mechanical signal transfer through the human body.

### 2.1. On-Body Communication

An on-body communication system is by definition a communication system that enables body-attached devices to communicate with each other. A previous study categorized on-body communication methods on the basis of their signal transfer methods: simple circuit type, electrostatic coupling type, and waveguide type [6].

#### 2.1.1. Simple Circuit Type

This type utilizes the human body as an electrical conductor in which electrical currents flow between the devices through the body. The operating principle is also used in bioelectrical impedance analysis. Although the electrical circuit including the body part is simple and straightforward to implement, this type requires systems to have an external wire that acts as a return path; therefore, it is difficult to implement this type for real-world applications, as pointed out in [6]. In addition, it requires electrodes to be robust against external factors, such as sweat/humidity and debris.

#### 2.1.2. Electrostatic Coupling Type

In an early pioneer study, Zimmerman *et al.* proposed the notion of PAN based on electrostatic coupling [1,7]. This type of near-field electrostatic coupling uses the human body to radiate an electric field [1,7,8]. In a previous study [7], an entire human body that emits low-frequency energy is technically defined as “the human transmitter.” The remote receiver device measures the signal from the transmitter wirelessly in the form of displacement currents. Such capacitive coupling between devices through the human body has a strong advantage in terms of encumbrance in that it does not require any devices to be physically wired for communication. Taking advantage of the jamming avoidance mechanism used by some species of weakly electric fish [9] in which transmission frequency shifts autonomously such that each fish uses a unique frequency [7], they further proposed the concept of multiple PANs that can operate together even in the same sensing space. An early work extended the concept of electrostatic coupling to a system that localizes the multitouch position [10]. In their system called *DiamondTouch*, target signals are transmitted through capacitive coupling between electrodes and human body parts. For example, signals from the antenna underneath the insulating layer of a display screen are transmitted to the finger via capacitive coupling. At the receiver side, which shares the electrical ground reference with the transmitter, the user is also capacitively coupled to the receiver antenna embedded in the surroundings (e.g., a chair). This coupling completes the proposed capacitively coupled circuit by sending the received signal back to the system.

As described, the approaches based on electrostatic coupling have advantages in that a human can be a part of the electrical circuit without any physical wires. However, one major drawback of this approach is that its communication quality is dependent on the surroundings, such as metals and human bodies, as pointed out in [6].

#### 2.1.3. Waveguide Type: Radio Waves

This type uses the human body as a waveguide, a structure that conveys signals. Compared to the electrostatic coupling type, the transmission quality is not affected by the surroundings. A previous study utilized the human body as a waveguide for electromagnetic waves for the purpose of intra-body communication [6]. The transmitter and receiver systems communicate through frequency-modulated electric currents of approximately 10.7 MHz. As an application example, they proposed a health care/medical application in which measured health data at the wristwatch, such as peripheral capillary oxygen saturation and heart rate, are transmitted to external touch devices.

Thus far, we have discussed on-body communication mainly focusing on electrical/electromagnetic signals. In the present study, we propose a mechanical waveguide-based communication system, which is similar to [6], except that our approach uses mechanical signals. To the best of our knowledge, this is the first proposal of a communication between a smartwatch and a touched devices based on mechanical signals through human body parts (*i.e.*, ultrasonic waves through skin/bone.) To better understand the characteristics of mechanical signals through the body, we briefly review the related work in the following section.

### 2.2. Mechanical Signal Transfer through Human Body Parts

There have been numerous application systems for transmitting acoustic signals through human body parts [11,12,13,14] by utilizing the fact that ultrasonic waves can be propagated through the human body (e.g., skin and bone [15] ). As a previous study described, it is worth noting that human body, which is composed of around 65% of waters, is not good medium for transmitting the RF electromagnetic waves [16].

In [14], a recent work proposed an ultrasonic transmission and a multiple access technique in the context of intra-body area networks. They validated the feasibility of the proposed method by experimentally demonstrating a communication between two nodes through a human-kidney phantom. In their follow-up study [17], they further proposed U-Wear architecture for enabling wearable medical devices to efficiently communicate with each other based on the near-field ultrasonic sounds. In another recent study, bone-conducted sound signals were utilized for measuring the touched position and angle of the elbow joint [11]. In their proposed passive sensing scenario, two microphones, located at the styloid process of the ulna and olecranon to capture bone-transmitted acoustic signals in a robust way, are used to measure the signal generated by touch. On the basis of the fact that the amplitude of the two signals is dependent only on the distance from the source (*i.e.*, finger taps) to the sensor, the system is able to estimate the touched position. In the active sensing scenario, two sensors measure reflected sinusoidal signals of 800 Hz, which are transmitted from the attached speaker, and analyze the magnitude of the signal. Considering that the amount of reflection is proportional to the elbow angle (*i.e.*, the more the elbow is bent, the less the signal is transmitted to the upper arm), they could estimate the elbow's bending angle on the basis of the measured signal intensity. Recently, Mujibiya *et al.* proposed a touch sensing system based on transdermal ultrasound propagation [12]. In one configuration, a finger-mounted receiver measures ultrasonic vibration transmitted from an armband-mounted transmitter on the contralateral arm. By utilizing frequency-based coding (35–50 kHz), the system could identify which body parts (e.g., forearm, anterior and posterior) are currently being touched. In addition, measured intensity is used for calculating the touched distance from the armband. Another recent project used the human body for transmitting acoustic signals for the purpose of resolving the location of finger taps on the forearm [13]. Their novel idea is mainly focused on utilizing the infinitesimal mechanical waves produced by finger impacts on skin for estimating the location of the on-body finger taps.

### 2.3. Communication between Devices with Mechanical Signals

In a previous study, an acoustic-based input technique was proposed to turn a wide variety of everyday objects, such as walls and tables, which can transmit sounds, into interaction surfaces [18]. This type of interaction is advantageous in that it can be seamlessly integrated into the regular environments. Another recent work has demonstrated the feasibility of mechanical vibration for inter-device communication purposes [19]. They achieved short-range wireless communication between two mobile devices by utilizing the built-in vibrating motor for actuation and an accelerometer for sensing. Compared to other types of near-field wireless communication methods, the proposed system has advantages in terms of scalability (*i.e.*, application to objects without radio functionalities).

## 3. Proposed System

**Figure 1:**
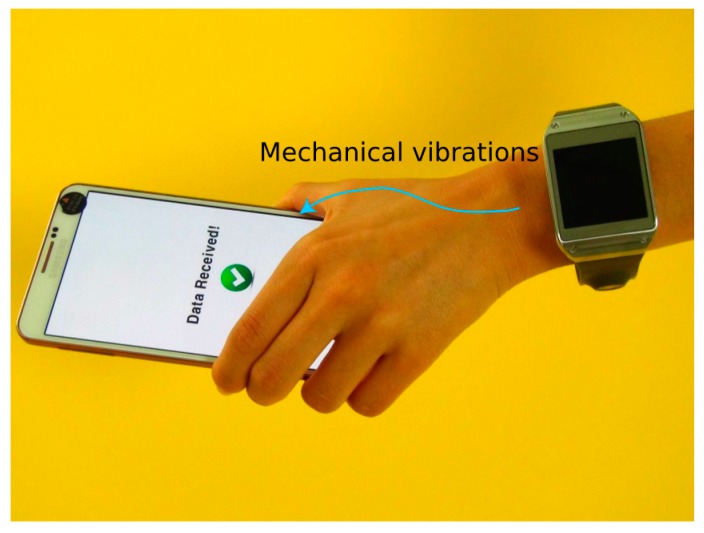
Proposed system that transmits data from a smartwatch to a touch device by modulating high-frequency mechanical waves. The signal is mechanically generated by the watch-mounted bone-conductor transducer.

Inspired by bone conductive transducers that deliver audio signals through the bones of the skull, our system leverages body-transmitted acoustic signals for enabling inter-device communication. That is, instead of the auditory ossicles, our proposed system guides the tiny vibrations to the electronic sensor of the receiver device through the human hand structure. The proposed interaction scheme is illustrated in Figure 1.

### 3.1. Hardware

**Figure 2:**
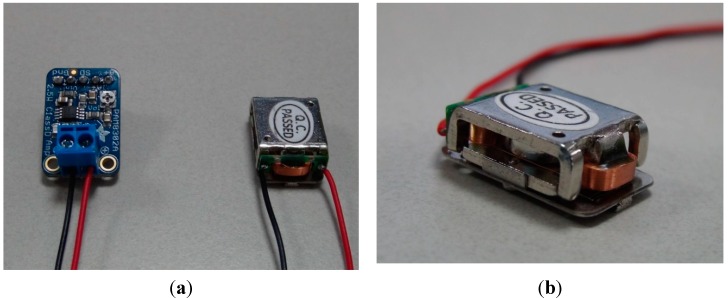
(**a**) The amplifier (left) and the surface transducer (right) used for the system; (**b**) a close view of the surface transducer.

**Figure 3:**
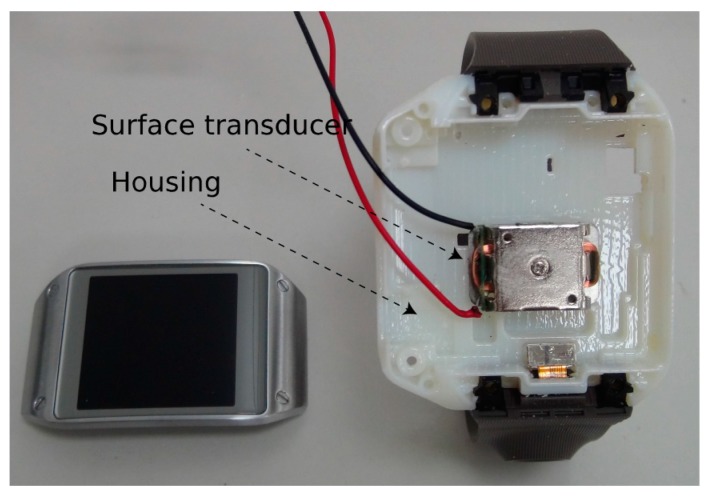
Internal view of the proposed system. A surface transducer is firmly mounted to the custom Galaxy Gear I housing.

We developed a pair of proof-of-concept devices that includes a transmitter and a receiver device. Throughout the paper, we limit our attention to a smartwatch worn on the wrist as a transmitter and a smartphone touched by the finger of the same hand as a receiver. In the transmitter device, a commercial surface transducer was embedded in an off-the-shelf smartwatch (Galaxy Gear I) for the purpose of generating the mechanical signals. The surface transducer, which does not make acoustic signals by itself because it does not have any moving parts (*i.e.*, cones), creates a tiny expansion/contraction of the metal parts to make the attached surface mechanically vibrate. When the transducer is attached to the skin around the ear, the generated tiny vibration is transmitted to the inner ear through the bones of the skull. In our case, the vibrations from the transducer are intended to flow to the tips of the fingers through the hand structure, which acts as a waveguide. To amplify the electrical signals sent to the transducer, we incorporated a 2.5-W Class-D mono audio amplifier, PAM8302A, by Diodes Incorporated. Figure 2 shows the amplifier and surface transducers used in our system, and Figure 3 shows the developed system. A data acquisition system (NI-6343 by National Instrument) was used for input signal generation.

At the receiver side, a piezoelement, originally used as a sound pickup, was attached to the front side of the smartphone to collect the transmitted vibrations. The same module of the data acquisition system, but with an isolated electrical ground, was employed to capture and analyze the collected signals.

### 3.2. Data Acquisition and Analysis

**Figure 4:**
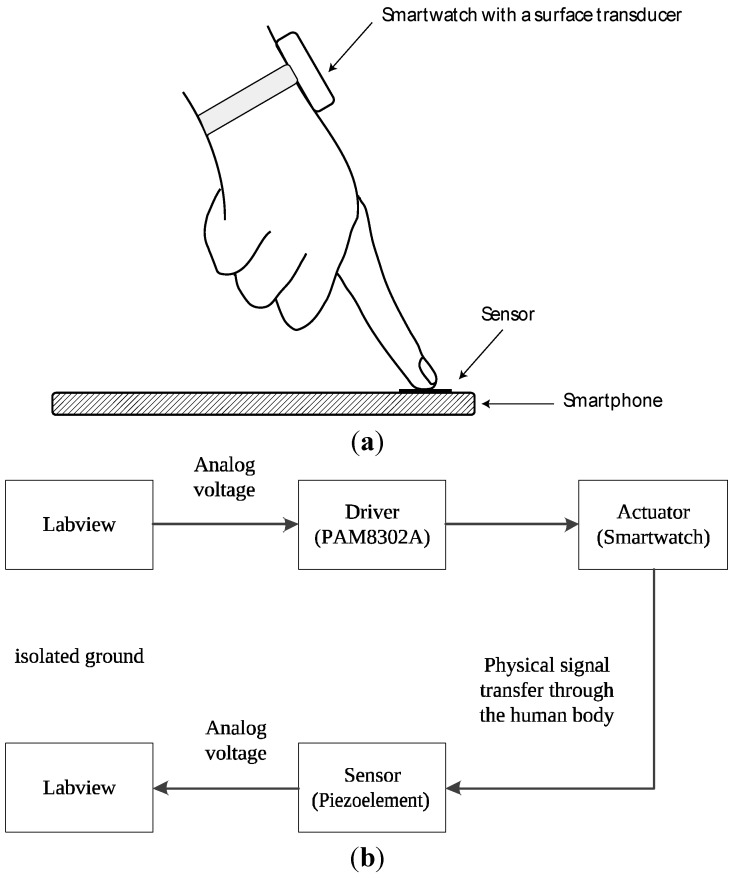
(**a**) Schematic of the measurement condition. A piezoelement, electrically insulated using insulation tape, was attached to the front side of the smartphone for capturing the incoming signal from the user's index finger; (**b**) System architecture of the proof-of-concept device.

We tested the proof-of-concept device with high-frequency signals. As Figure 4 shows in a schematic of the experimental condition, mechanical vibration transferred through the hand is captured by the piezo sensor attached on top of a smartphone. For the actuation signals, a set of rectangular waves was adopted, with frequencies of 18 kHz to 20 kHz at increments of 0.5 kHz. Each set of signals for driving the surface transducer was generated at the rate of 48 kHz using the data acquisition device. The voltage amplitude of the rectangular waveform was set to 0.5 V, and the measured average power consumption of the surface transducer was approximately 200 mW. The signals transmitted to the smartphone were collected at a rate of 96 kHz using a referenced single-ended (RSE) measurement of the data acquisition device. Analysis of the collected signals in the frequency domain was performed in LabView-based software.

### 3.3. Results and Discussion

**Figure 5:**
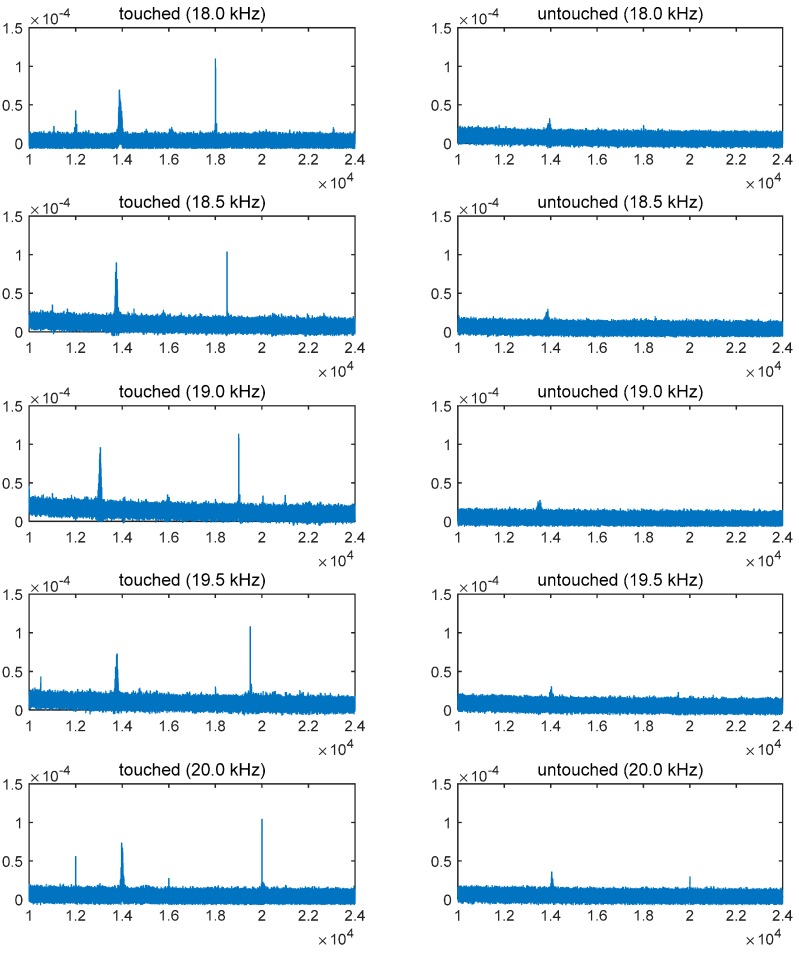
FFT analysis of the measured signals transferred from the smartwatch.

As shown in Figure 5, which shows the FFT analysis of the sensed signals at the smartphone side, the intended signals (18.0–20.0 kHz) were successfully captured in the receiver device. Airborne transmission of the signals was barely captured (see the right-hand side parts of Figure 5), meaning that signal transmission through the air is negligible. In an effort to assess the feasibility of the proposed method, we also measured the bit error rate (BER) with a modulation scheme of binary frequency-shift keying (BFSK). A fixed space frequency of 18 kHz and two different mark frequencies of 25 Hz and 50 Hz were employed in this setup. The measured BERs from the transmission of 1024 bit were 0.008 for the pair of (18,000 Hz and 18,025 Hz) and 0.003 for the pair of (18,000 Hz and 18,050 Hz.)

Although the experimental results show the feasibility of the proposed method, we have remaining issues. First, because we employed a commercial bone-conducting speaker whose structure is optimized for delivering audible sounds (up to approximately 20 kHz) as an actuation unit, high-frequency signals were not efficiently actuated. This issue needs to be considered due to the unavoidable signal attenuation in the medium between the transmitter and receiver. In case of the touched condition (see Figure 5, left-hand side), power differences measured at the actuator and the sensor were 21.29 dB for 18.0-kHz signal, 21.32 dB for 18.5-kHz signal, 21.17 dB for 19.0-kHz signal, 21.40 dB for 19.5-kHz signal, and 21.41 dB for 20.0-kHz signal, respectively. Second, undesirable low frequency signals, which may generate audible noise sounds, need to be removed although these do not harm the communication quality. Both of these issues can be fundamentally mitigated by developing a high-frequency dedicated bone-conducting transducer.

## 4. Applications

### 4.1. Dual Frequency Actuation

**Figure 6:**
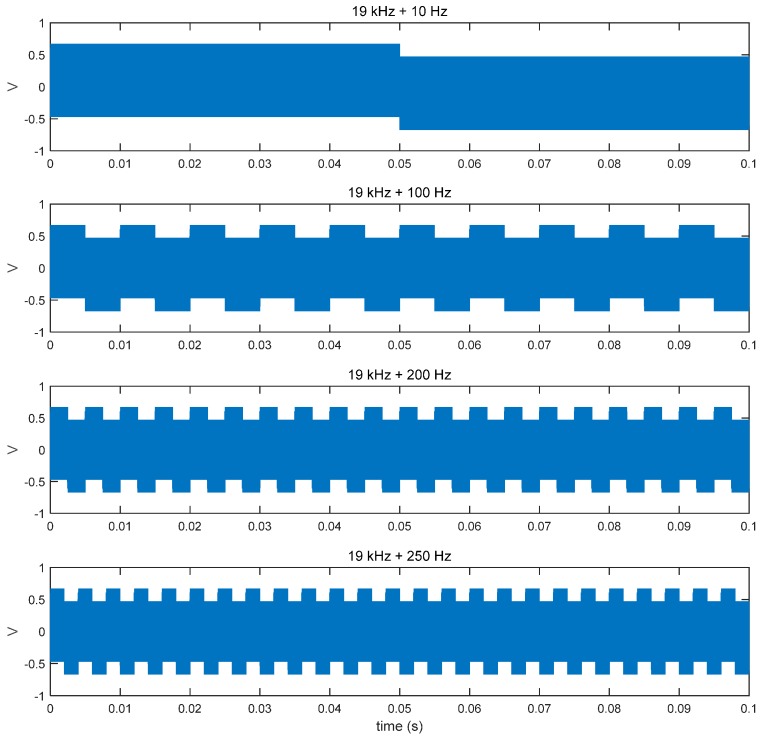
Input signals with additional low frequency vibrations for haptic effects.

As an application, we propose a dual-frequency actuation in a single surface transducer, with a high-frequency wave for data transmission (above 18 kHz) and low-frequency wave (up to 250 Hz) for haptic effects. To validate this approach, we measured output signals with additional low-frequency vibrations, as described in Figure 6. Both of the signals are generated on the basis of rectangular waves. The amplitude of the data signals was set to 0.5 V, and that for the haptic effects was set to 0.1 V. Power consumption was measured as approximately 280 mW (a 40% increment compared to the data-only transmission) when low-frequency haptic signals are actuated together with the data signals; an input voltage of 5 V was used for the audio amplifier.

**Figure 7:**
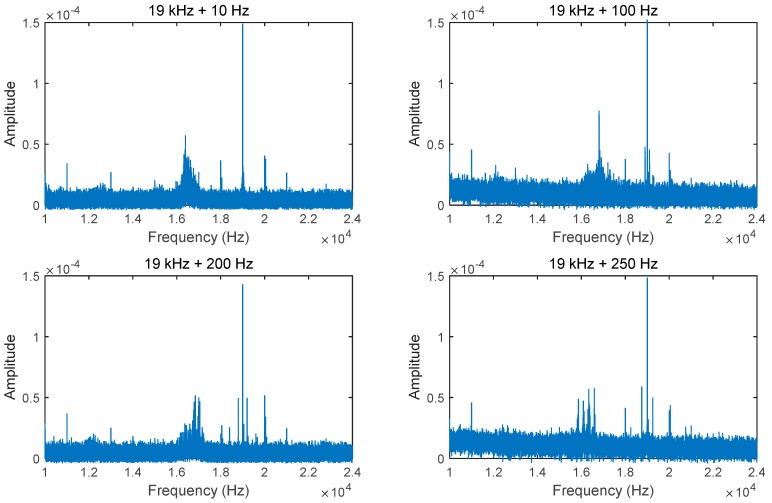
Results with additional low frequency vibrations.

Figure 7 shows that the intended high frequency signals (19 kHz) were successfully captured. Although additional noises are observed, they do not dominate over the target frequencies, meaning that the actuation of two signals does not affect the system performance. In summary, haptic effects can be designed into an application to give users the sense of actual information flow.

### 4.2. Encrypted Mechanical Signals

As the number of devices belonging to a user increases, transferring authentication to other related devices becomes important. A recent project called *SlickLogin* [20] has proposed a natural SSO (single sign-on) system to reduce the number of interaction between user and mobile devices based on inaudible ultrasonic sound. In their proposed scenario, a mobile phone can be automatically signed in once it listens to the inaudible sounds that contain authentication data from a PC on which the user is already signed in.

**Figure 8:**
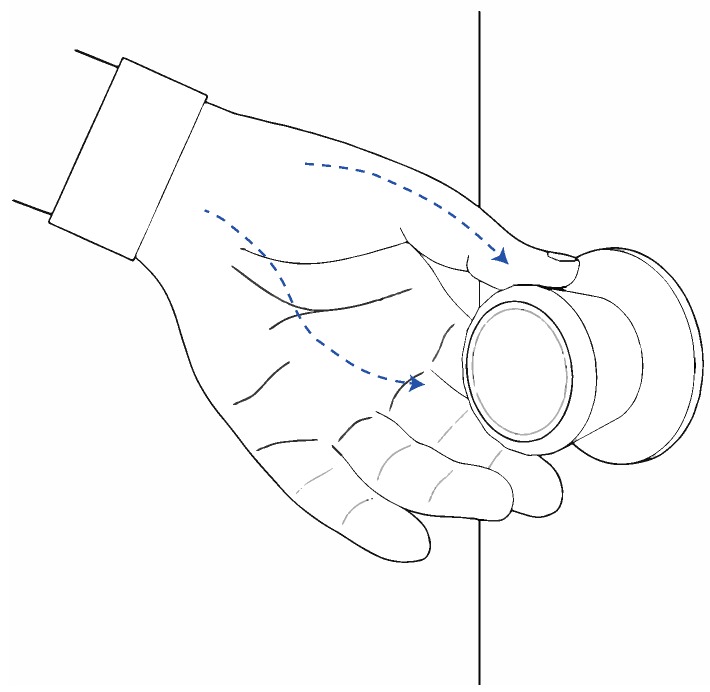
Application to a doorknob with a vibration sensor embedded. Either original or encrypted vibration can be transferred, depending on the required security level.

In a similar manner, our system can unlock a touch device or cause it to seamlessly sign in to a specific application once it receives authentication data from the smartwatch. Figure 8 illustrates this scenario in which smartwatch leverages physical contact between a user’s hand and a doorknob for realizing the function of unlocking the door. Without loss of generality, this configuration can be scaled to generic IoT devices.

Although this method has advantages in terms of keeping data confidentiality in that signals are not revealed to the air, this approach has one fundamental drawback: *signals can be captured on the way to the sensor*. In other words, a hacker can capture, decode, and emulate the signals. This issue can be handled by adopting the concept of a one-time password that hashes a message, *m_1_*, along with time information, *m_2_*, which results in time-varying encoded signals, *c*. With this approach, the actuation signals, e.g., *c* = SHA(*m_1_* + *m_2_*), may be captured by attackers, but cannot be decoded or used at a different time. Although the encryption can be further secured using a variety of cryptographic methods such as public-key cryptography, this is beyond the scope of this study.

## 5. Conclusions

In this paper, we have proposed a mechanical-based sensor and actuation system. As an actuation unit in the sending device, we utilized a commercial surface transducer, originally used as a bone conduction speaker, for generating mechanical vibrations in the attached surface, *i.e.*, skin. Experimental results show that the proposed system could successfully deliver intended signals to the sensor through the human hand structure. We believe that the proposed approach can be scaled to a variety of wearable and IoT applications that need secure communication between devices in that most of the signals are transferred through the physical structure of the human body. We also envisioned the concept of dual-frequency actuation under which a high-frequency data signal is actuated along with a low-frequency haptic signal with additional low frequency vibrations (*i.e.*, 10, 100, 200, and 250 Hz), and validated its feasibility by measuring the output signals. Our future plans include developing a surface transducer that is dedicated to high-frequency (e.g., above 20.0 kHz) signal actuation to generate the intended vibrations more efficiently. We also plan to develop haptic flow effects with low-frequency vibrations (up to 250 Hz) and incorporate them into the present system such that users can be aware of information flow.

## References

[1] Zimmerman T.G. (1996). Personal Area Networks: Near-field intrabody communication. IBM Syst. J..

[2] Mulliner C. Vulnerability Analysis and Attacks on NFC-Enabled Mobile Phones. Proceedings of the 2009 International Conference on Availability, Reliability and Security.

[3] Ahlbom A., Bergqvist U., Bernhardt J.H., Cesarini J., Court L.A., Grandolfo M., Hietanen M., Mckinlay A.F., Repacholi M.H., Sliney D.H. (1998). Guidelines for limiting exposure to time-varying electric, magnetic, and electromagnetic fields (up to 300 GHz). Health Phys..

[4] Wegmüller M.S. (2007). Intra-Body Communication for Biomedical Sensor Networks. Ph.D. Thesis.

[5] Webster J.G. (1978). Medical Instrumentation-Application and Design. J. Clin. Eng..

[6] Hachisuka K., Nakata A., Takeda T., Terauchi Y., Shiba K., Sasaki K., Hosaka H., Itao K. Development and performance analysis of an intra-body communication device. Proceedings of the 12th International Conference on TRANSDUCERS, Solid-State Sensors, Actuators and Microsystems.

[7] Zimmerman T.G., Smith J.R., Paradiso J.A., Allport D., Gershenfeld N. Applying electric field sensing to human-computer interfaces. Proceedings of the 1995 SIGCHI Conference on Human Factors in Computing Systems.

[8] Cohn G., Morris D., Patel S.N., Tan D.S. Your noise is my command: Sensing gestures using the body as an antenna. Proceedings of the 2011 SIGCHI Conference on Human Factors in Computing Systems.

[9] Bullock H.T., Hopkins C.D., Fay R.R. (2006). Electroreception.

[10] Dietz P., Leigh D. DiamondTouch: A multi-user touch technology. Proceedings of the 14th Annual ACM Symposium on User Interface Software and Technology.

[11] Takemura K., Ito A., Takamatsu J., Ogasawara T. Active bone-conducted sound sensing for wearable interfaces. Proceedings of the 24th annual ACM Symposium Adjunct on User Interface Software and Technolog.

[12] Mujibiya A., Cao X., Tan D.S., Morris D., Patel S.N., Rekimoto J. The sound of touch: on-body touch and gesture sensing based on transdermal ultrasound propagation. Proceedings of the 2013 ACM International Conference on Interactive Tabletops and Surfaces.

[13] Harrison C., Tan D., Morris D. Skinput: Appropriating the body as an input surface. Proceedings of the 2010 SIGCHI Conference on Human Factors in Computing Systems.

[14] Santagati E.G., Melodia T. Sonar inside your body: Prototyping ultrasonic intra-body sensor networks. Proceedings of the 2014 IEEE INFOCOM.

[15] Van der Perre G., Lowet G. (1996). *In vivo* assessment of bone mechanical properties by vibration and ultrasonic wave propagation analysis. Bone.

[16] Santagati G.E., Melodia T., Galluccio L., Palazzo S. (2013). Ultrasonic networking for e-health applications. IEEE Wirel. Commun..

[17] Santagati G.E., Melodia T. U-Wear: Software-Defined Ultrasonic Networking for Wearable Devices. Proceedings of the 13th Annual International Conference on Mobile Systems, Applications, and Services.

[18] Harrison C., Hudson S.E. Scratch input: Creating large, inexpensive, unpowered and mobile finger input surfaces. Proceedings of the 21st Annual ACM Symposium on User Interface Software and Technology.

[19] Hwang I., Cho J., Oh S. (2014). VibeComm: Radio-Free Wireless Communication for Smart Devices Using Vibration. Sensors.

[20] GOOGLE INC. SlickLogin. http://www.slicklogin.com/.

